# On the trail of a tropical disease

**DOI:** 10.7554/eLife.01115

**Published:** 2013-07-30

**Authors:** Alejandro Sánchez Alvarado

**Affiliations:** 1 **Alejandro Sánchez Alvarado** is an *eLife* reviewing editor, and is at the Howard Hughes Medical Institute, Stowers Institute for Medical Research, Kansas City, United Statesasa@stowers.org

**Keywords:** *Schistosoma mansoni*, stem cells, neoblasts, trematode development, schistosomiasis, Other

## Abstract

Learning more about the cells that enable parasitic worms called schistosomes to reproduce inside snails could lead to new treatments that prevent these parasites from being transmitted to humans.

**Related research article** Wang B, Collins JJ III, Newmark PA. 2013. Functional genomic characterization of neoblast-like stem cells in larval *Schistosoma mansoni*. *eLife* **2**:e00768. doi: 10.7554/eLife.00768

**Image** Schistosome parasites (purple) undergo asexual reproduction inside a snail host

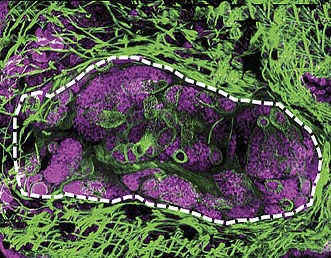

Diseases caused by parasites can have devastating effects, particularly in the developing world. Some of these effects are quantifiable, including morbidity and loss of life, while others are much harder to measure, such as the loss of human potential. The fact that children are especially vulnerable to infection makes these diseases particularly tragic. One such disease is schistosomiasis, also known as bilharzia after its discoverer, Theodor Bilharz (Jordan, 2000). It is caused by blood-dwelling parasitic flatworms called schistosomes, and is estimated to affect about 250 million people worldwide (WHO, 2011), with a further 800 million at risk of infection (Steinmann et al., 2006). Now, in *eLife*, Bo Wang, James Collins III and Phillip Newmark from the University of Illinois at Urbana-Champaign report new insights into the biology of schistosomes that could potentially lead to novel strategies for controlling their transmission (Wang et al., 2013).

Schistosomiasis is primarily caused by two species of parasites, *Schistosoma japonicum* and *Schistosoma mansoni*, which differ in prevalence across the world. The genomes of both species have been sequenced (Berriman et al., 2009; The Schistosoma japonicum genome sequencing and functional analysis consortium, 2009), and both infections can be treated with an efficient, widely available and relatively inexpensive drug, praziquantel. So why have we failed to eradicate schistosomiasis? Reasons abound, but the fact that efforts have focused primarily on pharmacological treatment of infected individuals means that socioeconomic factors—such as a lack of infrastructure, poor management of resources, and political instability in affected regions—have been able to thwart attempts to control the disease. It would be much better, therefore, if we could develop methods to combat schistosomes before they infect humans, rather than only treating people after the event.

The parasites that cause schistosomiasis are members of the phylum Platyhelminthes, and have a complex life cycle that alternates between sexual and asexual generations in two different hosts: mammals and snails. Eggs are first excreted from a mammalian host into freshwater, where they hatch into free-living larvae called miracidia. The larvae then infect snails, and undergo a remarkable asexual embryogenesis that results in sporocysts (Figure 1) laden with pluripotent stem cells called germinal cells, which then differentiate to produce parasites that can infect humans**.** The parasites, which are now known as cercariae, emerge from the snail into freshwater, where they can burrow through the epidermis of mammals. Once inside a host, they develop into adults and begin to reproduce sexually.

**Figure 1.**
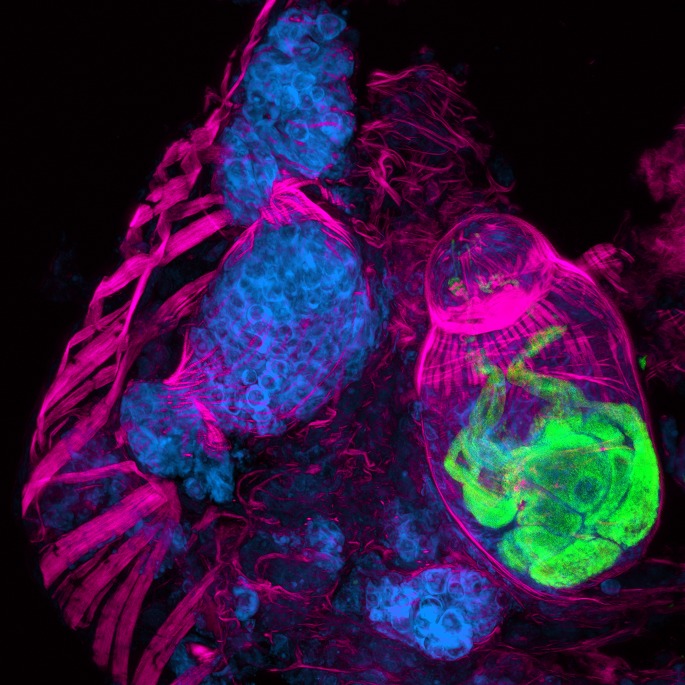
A cryosection of a *Schistosoma mansoni* daughter sporocyst within a snail intermediate host, six weeks post-infection. The section is stained with: phalloidin (magenta) to show the musculature in both the snail and schistosome; POPO-1 (blue) to label germinal cells in the sporocyst; and lectin PSA (green) to reveal secretory glands within the developing parasite embryo. The image is a maximum intensity projection of confocal sections.

IMAGE: BO WANG, JAMES COLLINS III, PHILLIP NEWMARK, HHMI/UNIVERSITY OF ILLINOIS AT URBANA-CHAMPAIGN.

Germinal cells within sporocysts have long been thought to be responsible for the asexual multiplication of the parasite within snails (Olivier and Mao, 1949). However, little to nothing is known about these cells at the molecular level. Wang, Collins and Newmark have now characterized the germinal cell population by profiling gene expression in the cells, validating these findings with fluorescence in situ hybridization, and then using RNA interference to silence individual genes in order to determine their functions.

Their results uncovered similarities, at both the molecular and functional level, between these germinal cells and stem cells that have recently been identified in adult parasites (Collins et al., 2013). There were also similarities with pluripotent stem cells known as neoblasts found in non-parasitic free-living flatworms (planarians), and with cells that produce gametes in other multicellular animals. For instance, a subpopulation of germinal cells express a gene called *nanos-2*, which is involved in regulating gamete production across multiple taxa. Germinal cells that express this gene progress more slowly through the cell cycle—consistent with the conserved role of *nanos* in lengthening the cell cycle by repressing mitosis in the primordial germ cells of many animals.

Finally, the work of Wang, Collins III and Newmark unites two important, but disparate, fields of biomedical science—parasitology and developmental biology—and demonstrates the potential for these types of analyses to enrich and inform both. Studying an organism with a genome that essentially encodes multiple body plans (miracidia, sporocysts, cercariae, plus different forms of the adult in the blood, lungs and liver of mammalian hosts) should enable us to uncover vulnerabilities in development that will provide opportunities to intervene and block disease transmission. Attacking the parasite when it is outside the human body removes issues associated with human physiology and should significantly mitigate socioeconomic problems that impede the delivery of drugs. Imagine eradicating the risk of schistosomiasis for 800 million people, not by treating them, but by treating the snails in their water supply instead. By changing the way we attempt to eradicate parasitic diseases, this approach could ultimately improve the health of millions.
